# Stochastic rounding: implementation, error analysis and applications

**DOI:** 10.1098/rsos.211631

**Published:** 2022-03-09

**Authors:** Matteo Croci, Massimiliano Fasi, Nicholas J. Higham, Theo Mary, Mantas Mikaitis

**Affiliations:** ^1^ Oden Institute, University of Texas at Austin, Austin, TX, 78712, USA; ^2^ Department of Computer Science, Durham University, Durham, DH1 3LE, UK; ^3^ Department of Mathematics, The University of Manchester, Manchester, M13 9PL, UK; ^4^ Sorbonne Université, CNRS, LIP6, Paris, 75005, France

**Keywords:** floating-point arithmetic, rounding error analysis, IEEE 754, binary16, bfloat16, machine learning

## Abstract

Stochastic rounding (SR) randomly maps a real number *x* to one of the two nearest values in a finite precision number system. The probability of choosing either of these two numbers is 1 minus their relative distance to *x*. This rounding mode was first proposed for use in computer arithmetic in the 1950s and it is currently experiencing a resurgence of interest. If used to compute the inner product of two vectors of length *n* in floating-point arithmetic, it yields an error bound with constant $\sqrt{n}u$ with high probability, where *u* is the unit round-off. This is not necessarily the case for round to nearest (RN), for which the worst-case error bound has constant *nu*. A particular attraction of SR is that, unlike RN, it is immune to the phenomenon of stagnation, whereby a sequence of tiny updates to a relatively large quantity is lost. We survey SR by discussing its mathematical properties and probabilistic error analysis, its implementation, and its use in applications, with a focus on machine learning and the numerical solution of differential equations.

## 1. Introduction

Rounding is the act of mapping a given number to one having a certain number of digits in a given base. For illustration, consider the task of rounding, in base 10, a 2-significant-digit number to 1 significant digit. If we round to the closest 1-digit number, for example, then 1.4 rounds to 1 and 1.7 rounds to 2. We denote the rounding operator by fl, thus we write fl(1.4) = 1 and fl(1.7) = 2. This rounding rule, called round to nearest (RN), is deterministic: the value of fl(*x*) depends only on *x*, and repeating the rounding yields the same result.

Suppose we want to compute 1 + 0.1 in 1-digit base-10 arithmetic. With RN we obtain fl(1 + 0.1) = 1. Another option is to round to either of the two nearest 1-digit numbers with a probability that depends on the distances to those numbers. If in our example we define fl(1 + 0.1) as 1 with probability 0.9 and as 2 with probability 0.1, then the expected result is 0.9 × 1 + 0.1 × 2 = 1.1, which is the exact answer. This probabilistic rounding is called *stochastic rounding* (SR), and this simple example demonstrates one way in which it can be useful in practice: by occasionally rounding up the value of a sum, SR avoids the phenomenon of stagnation, whereby a long sum of small quantities—0.1 in this example—is lost to rounding.

SR was first proposed over 60 years ago, but until recently had proved useful only in rather specialized contexts. In the last 5 years or so, however, this rounding mode has enjoyed a resurgence of interest, mainly because of the increasing availability of low-precision floating-point arithmetic in hardware and the recognition that, compared with RN, SR can produce errors that grow more slowly with the problem size. By its very nature, SR ensures that rounding errors are random and so encourages cancellation of errors, and while this effect benefits all precisions, it is particularly important at low precisions, where numbers have perhaps only 3 or 4 significant decimal digits and error growth can quickly destroy all accuracy. The worst-case error bounds for SR are a factor 2 larger than those for RN, however, and so SR does not benefit all computations.

The aim of this work is to survey SR, describing

— its basic properties (§2),
— its history (§3),
— floating-point arithmetics to which it might be applied (§4),
— how SR compares with RN as regards its basic properties, including ways in which SR is less satisfactory than RN (§5),
— its probabilistic rounding error analysis (§6),
— how it can be implemented (§7), and
— how and why it is being used in applications (§8).

## 2. What is stochastic rounding?

Let *F* denote a finite subset of R. We denote by fl any rounding operator that maps numbers in R to either of the two nearest numbers in *F*. For x∈R, define the two rounding candidates

⌊x⌋=max{y∈F : y≤x}and⌈x⌉=min{y∈F : y≥x},

so that ⌊x⌋≤x≤⌈x⌉, with equality throughout if *x* ∈ *F*. Note that when x∉F, the two numbers ⌊x⌋ and ⌈x⌉ are adjacent in *F*.

For x∈R∖F, SR is defined by

2.1 $$\text{fl}(x)=\left\{ \begin{array}{rl} \lceil x\rceil ,\; & \;\text{with probability}\;q(x),\;\\ \lfloor x\rfloor ,\; & \;\text{with probability}\;1-q(x), \end{array}\right.$$

where *q*(*x*) ∈ [0, 1]. The simplest choice is *q*(*x*) = 0.5, in which case we round up or down with equal probability, independently of *x*. As is customary in the literature [1,2], we call this less commonly used form of SR *mode 2 SR*. Another choice is to set in (2.1)

**Figure 1.**
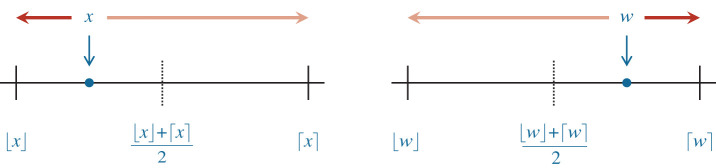
Stochastic rounding rounds the real number *x* to the next smaller or the next larger value in *F*, which we denote by ⌊x⌋ and ⌈x⌉, respectively. In the example on the left, *x* is one quarter of the way between ⌊x⌋ and ⌈x⌉, thus RN will round *x* to ⌊x⌋, while mode 1 SR will round it to ⌈x⌉ with probability *q*(*x*) = 0.25 and to ⌊x⌋ with probability 1 − *q*(*x*) = 0.75. In the example on the right, *w* is three-quarters of the way between ⌊w⌋ and ⌈w⌉, thus RN will round *w* to ⌈w⌉, while mode 1 SR will round it to ⌈w⌉ with probability *q*(*w*) = 0.75 and to ⌊w⌋ with probability 1 − *q*(*w*) = 0.25.

2.2 $$q(x)=\frac{x-\lfloor x\rfloor }{\left\lceil x\rceil -\lfloor x\right\rfloor },$$

which means that we round *x* to the next larger or smaller number *y* ∈ *F* with probability 1 minus the distance between *x* and *y* divided by ⌈x⌉−⌊x⌋. See figure 1 for an illustration. The choice (2.2) yields *mode 1 SR*, which is the most interesting SR mode from a numerical point of view. Unless otherwise stated, here SR means mode 1 SR.

For the rest of this paper, we take *F* to be a floating-point number system, unless otherwise stated, as this is the case of greatest interest, but much of what we say is applicable to fixed-point arithmetic.

## 3. Early history of stochastic rounding

To the best of our knowledge, the earliest proposal of SR was in a one-paragraph abstract of a communication presented by Forsythe in 1949 at the 52nd meeting of the American Mathematical Society [3]. The abstract claims that SR can be used to reduce the accumulation of round-off errors observed by Huskey [4] in solving a simple system of ordinary differential equations (ODEs). The numerical integration that Forsythe and Huskey consider entails a sum of real values which is further reduced to a sum of integers, most likely intended as fixed-point representations of reals. The suggestion is to perform this rounding by *random round-off*, a suggestive name for mode 1 SR. The abstract concludes by stating that numerical tests on some unspecified IBM equipment confirm that SR can eliminate the ‘peculiarities’ noticed by Huskey on the ENIAC.^1^

The first hardware implementation of SR we are aware of was described by Barnes *et al.* [5] in 1951. The authors describe a digital computer with 8-digit decimal arithmetic and explain that using SR rather than RN in multipliers and dividers simplified the implementation. As their implementation rounds up or down with equal probability, this constitutes an early example of mode 2 SR.

A note by Forsythe, originally written in 1950 and reprinted in 1959 [6] (see [6], footnote 1), provides more details about the proposal to round stochastically when solving ODEs. The document suggests to implement mode 1 SR for decimal arithmetic as follows:

*On a decimal machine, instead of adding a 5 in the most significant position of the digits to be dropped (ordinary rounding off), one adds a random decimal digit to each of the digital positions to be dropped. As with ordinary rounding off, the addition carry-over determines whether the rounding off is ‘up’ or ‘down’.*

It is not clear whether this excerpt refers to a hardware implementation or to a modification that could be done in software on the computers of the time. This technique has been used in recent hardware implementations for rounding binary numbers [7,8].

In a 1966 paper, Hull & Swenson [9] test various probabilistic rounding error models by comparing the results of stochastically rounded operations with the expected error predicted by the models. According to the description provided at the beginning of the section ‘Simulation of the Models’ [9, p. 109], however, the implementation of SR that Hull & Swenson consider differs from the one we examine. In order to round stochastically the result of an arithmetic operation, they first perform the operation in double precision arithmetic, then add a pseudo-random number between −1/2 and 1/2 of the unit in the last place of the most significant half of the double precision result. Subsequent calculations use the modified double precision value, which presumably includes the original quantity in its least significant half and the added random quantity. Despite the different spirit, we mention this contribution here as it is one of the earliest manuscripts we are aware of that considers non-deterministic rounding modes.

## 4. Floating-point arithmetics

Before describing the finer details of SR, we recall some necessary background on floating-point arithmetic. We discuss the formats in the IEEE 754 standard for floating-point arithmetic and two other formats of practical interest, bfloat16 and TensorFloat-32.

### 4.1. IEEE 754 standard floating-point arithmetics

The IEEE standard 754 for floating-point arithmetic was first released in 1985 [10] and then revised in 2008 [11] and 2019 [12]. The standard dictates the encoding rules for binary and decimal floating-point data types, the precision and exponent range of some standard formats, and the accuracy requirements of basic arithmetic operations. It also prescribes how to handle exceptional cases and specifies a set of recommended mathematical functions that software and hardware floating-point libraries should provide in order to ensure a consistent numerical behaviour. Table 1 reports the parameters for four binary floating-point data types defined in the latest revision of the standard. Most hardware implements the data types binary32 and binary64, commonly known as *single* and *double precision*, respectively. Of the remaining formats, binary16 is defined only as a storage format, but it has been implemented as an arithmetic in hardware by several manufacturers. One other IEEE format is binary128, which is not listed in the table. While binary128 is mainly supported in software, it is also available in hardware on the IBM Power9 [13] and z13 [14] processors.

**Table 1.** Parameters of various binary floating-point formats: number of digits of precision including the implicit bit (*p*), smallest positive representable normal (*f*_min_) and subnormal (*s*_min_) numbers, and largest positive number (*f*_max_). The ‘binary*xy*’ formats are from the IEEE 754 standard.

	bfloat16	binary16	binary32	binary64
*p*	8	11	24	53
*f*_min_	2^−126^	2^−14^	2^−126^	2^−1022^
*s*_min_	2^−133^	2^−24^	2^−149^	2^−1074^
*f*_max_	2^127^(2 − 2^−7^)	2^15^(2 − 2^−10^)	2^127^(2 − 2^−23^)	2^1023^(2 − 2^−52^)

We now briefly recall some key aspects of IEEE floating-point number systems and the definitions and main properties of *normalization* and *subnormal numbers*. We focus on binary formats, since most commercially available hardware implements only binary arithmetic. A binary floating-point number *x* has the form

$${\left(-1\right)}^{s}\times m\times 2^{e-p+1},$$

where *s* is the sign bit, *p* is the *precision*, *m* ∈ [0, 2^*p*^ − 1] is the integer significand, and *e* ∈ [*e*_min_, *e*_max_], with *e*_min_ = 1 − *e*_max_, is the integer exponent. In order for *x* ≠ 0 to have a unique representation, the number system is *normalized* so that the most significant bit of *m*—the *implicit bit* in IEEE 754 parlance—is always set to 1 if $|x|\geq 2^{e_{\min}}$. Therefore, all floating-point numbers with *m* ≥ 2^*p*−1^ are normalized. Numbers that have absolute value below that of the smallest normalized number $2^{e_{\min}}$ are said to be *subnormal*: they have exponent *e* = *e*_min_, integer significand *m* < 2^*p*−1^, and therefore precision lower than that of normalized values (between 1 and *p* − 1 bits). Subnormal numbers provide the means to represent values in the *subnormal range* $\left(-2^{e_{\min}},2^{e_{\min}}\right)$, and are necessary in order to ensure that a floating-point number system satisfies Sterbenz’s lemma (described in §5) and has desirable properties such as gradual underflow. Because of the variable precision, however, subnormal numbers require special treatment in both software and hardware implementations of floating-point arithmetics. This is likely to cause performance and chip area overhead, and as a result it is not uncommon for hardware manufacturers not to support subnormal numbers. Two important numbers related to the precision *p* are the *machine epsilon*

4.1 $$\varepsilon_{M}=2^{1-p},$$

which is the spacing of the floating-point numbers just to the right of 1, and the *unit round-off*

4.2 $$u=2^{-p}=\frac{1}{2}\varepsilon_{M},$$

which is an upper bound on the relative error incurred when a real value is rounded to a precision-*p* floating-point representation using RN. For further details, we refer the reader to [15, ch. 1] and [16, ch. 2].

The latest revision of the IEEE 754 standard defines six rounding modes, which are listed in table 2. Four rounding modes are required for a floating-point arithmetic to be compliant: round to nearest with ties to even (RN), round towards positive (or towards +∞, or up, RU), round towards negative (or towards −∞, or down, RD), and round towards zero (RZ). In the rest of the paper we refer to these modes collectively as ‘standard rounding’.

**Table 2.** Rounding modes defined in the 2019 revision of the IEEE 754 standard [12].

rounding mode	description
to nearest with ties to even (RN)	round to a nearest floating-point value and if the two nearest floating-point values are equally close, round to the one with an even least significant digit. This is a default rounding mode.
to nearest with ties to away	round to a nearest floating-point value and if the two nearest floating-point values are equally close, round to the number with larger magnitude. Only required for decimal floating-point data types.
to nearest with ties to zero	round to a nearest floating-point value and if the two nearest floating-point values are equally close, round to the number with smaller magnitude. Only required for *augmented operations* [12, sec. 9.5].
towards positive (RU)	round to a nearest floating-point value that is no less than the argument.
towards negative (RD)	round to a nearest floating-point value that is no larger than the argument.
towards zero (RZ)	round to a nearest floating-point value that is no larger in magnitude than the argument.

The IEEE 754-2019 standard recommends *extended or extendable precisions* [12, §3.7] to enhance the basic formats listed in table 1. As an example, Intel provides an 80-bit extended precision format that has a 15-bit exponent and a 64-bit significand—the bit to the left of the radix point being stored explicitly in this case, as opposed to the IEEE 754 formats, which rely on the implicit bit convention and use the value of the exponent field to determine the leading bit of the significand. Arithmetic operations can be performed in higher precision and the results need not be rounded to binary64 until the final result of a computation leaves the higher-precision registers. Note that the use of 80-bit arithmetic is susceptible to double rounding, whereby a value may be rounded incorrectly to the final floating-point format when it is rounded to an intermediate format (extended precision, in this case) first [17,18]. Boldo & Melquiond have shown, using the Coq proof assistant, that double-rounding issues can be avoided if the extended precision format uses a deterministic rounding mode called round to odd [19,20].

### 4.2. Non-IEEE arithmetics

Among the non-IEEE floating-point formats implemented in recent hardware, we are particularly interested in those based on binary32: bfloat16 and TensorFloat-32, which lower the precision *p* from 23 to 8 and 11 bits, respectively. The main idea behind these formats is to reduce the memory and hardware arithmetic costs without narrowing the dynamic range; this contrasts with the aim behind the binary16 format, which allocates to the exponent field fewer bits than binary32 and therefore has a more limited dynamic range.

Bfloat16, which was originally proposed by Google and formalized by Intel [21], is available on the Armv8 architecture [22], on the NVIDIA Ampere chips [23] and on some Intel microarchitectures [24].

TensorFloat-32 is a format used internally in the tensor cores (matrix multiply-accumulate units) of the NVIDIA Ampere microarchitecture [23]. This 19-bit format is meant to be a low-precision replacement for binary32, but is not used for data storage and is not available in any other arithmetic unit on these GPUs.

## 5. Stochastic rounding versus round to nearest

In RN, which is the default rounding mode in most floating-point arithmetics, fl(*x*) is the number in *F* nearest to *x*, with some tie-breaking strategy for handling the case where *x* is equidistant from the next and previous floating-point numbers. While SR and RN share some properties, they also differ in some important respects. We first describe three properties that SR and RN have in common. In fact, the first two properties hold for any rounding mode.

— If *x* ∈ *F* then fl(*x*) = *x*, that is, rounding a floating-point number leaves it unchanged.
— If *x* and *y* are floating-point numbers with *y*/2 ≤ *x* ≤ 2*y* then fl(*x* − *y*) = *x* − *y* (assuming subnormal numbers are supported). This result, known as Sterbenz’s lemma [15, thm. 2.5], [25, thm. 1.8.2], relies on the fact that *x* − *y* is exactly representable.
— In base-2 arithmetic, any floating-point numbers *x* and *y* such that *x* ≤ *y* satisfy the inequalities
  $$x\leq \frac{\text{fl}(x+y)}{2}\leq y,$$
  which do not hold for all bases [1, §3.1].

SR and RN differ in some key properties, however.

For example, if x∉F then in general fl(|x|)≠|fl(x)| and fl( − *x*) ≠ −fl(*x*) for SR. Moreover, SR is not monotonic: *x* < *y* does not imply that fl(*x*) ≤ fl(*y*), as can be seen by considering any pair of reals *x* and *y* lying between two adjacent floating-point numbers.

Several results that describe how simple identities for real numbers are (partially) preserved for floating-point numbers under RN are well known. Generally similar, but weaker, results hold for SR. For a binary format with RN, for example, we have that $\mathrm{fl}(\sqrt{x^{2}})=|x|$ if *x* is a floating-point number [15, Prob. 2.20], barring underflow and overflow. For SR, however, if *x* is a floating-point number in the interval (1, 2) then $\mathrm{fl}(\sqrt{x^{2}})\in \{|x|-\varepsilon_{M},|x|,|x|+\varepsilon_{M}\}$, where *ɛ*_*M*_ is the machine epsilon defined in (4.1). A consequence of this fact is that the inequality $\mathrm{fl}(x/\sqrt{x^{2}+y^{2}})\leq 1$, which is always satisfied by RN [15, Prob. 2.21], is not necessarily true when SR is used.

Kahan proved that if *m* and *n* are integers such that $|m|<2^{p-1}$ (where *p* is the precision) and *n* = 2^*i*^ + 2^*j*^ for some *i* and *j* then fl(*n* × fl(*m*/*n*)) = *m* with RN [26, thm. 7]. Thus for example, fl(5 × fl(*m*/5)) = fl(17 × fl(*m*/7)) = *m*. Under SR, however, we can say only that fl(*n* × fl(*m*/*n*)) is either *m*, the next smaller floating-point number, or the next larger floating-point number.

Full details of the above results, as well as other properties that differ between SR and RN, are given by Connolly *et al*. [1, §3]. Before replacing RN with SR it is vital to consider whether a certain computation relies on properties of RN that go beyond the standard model of floating-point arithmetic (6.2) below, and if so, whether these properties remain true for SR. The solution of the quadratic equation *ax*^2^ + *bx* + *c* = 0 is a striking example of the subtle issues that may occur when switching from RN to SR: if evaluated using SR, the discriminant *b*^2^ − 4*ac* can be negative even when *b*^2^ > 4*ac*. This is a consequence of the non-monotonicity of SR, and it could lead one to incorrectly conclude that a quadratic equation has no real solutions when in fact it has two (almost) identical real roots.

The results in this section suggest that SR is less attractive than RN. It is the rounding error results described in the next section that account for the interest in SR.

## 6. Rounding error analysis with SR

If x∈R lies within the range of the floating-point number system *F*, it can be shown that for RN one has

6.1 fl(x)=x(1+δ),|δ|<u,

where the unit round-off *u* is defined in (4.2). Rounding error analysis is usually based on the standard model of floating-point arithmetic [15, eqn (2.4)], which assumes that the elementary arithmetic operations are rounded to nearest (as is the case for IEEE standard arithmetic with the default rounding mode), so that if no overflow or underflow occurs they satisfy

6.2 fl(x op y)=(x op y)(1+δ),|δ|≤u, op∈{+,−,×,/}.

Analogous models can be devised for unitary operations, such as the square root, or ternary operations, such as the fused multiply-add.

The model (6.2) is customarily used in rounding error analysis, and is based on the premise that the result of a floating-point elementary arithmetic operation should be as accurate as the correctly rounded infinitely precise result. Error analysis based on (6.2) is referred to as worst-case analysis because it can only use |*δ*| ≤ *u* and cannot exploit possible cancellation of rounding errors across multiple operations or instances where *δ* = 0.

For RN, the bound in (6.1) can be tightened to |*δ*| ≤ *u*/(1 + *u*) < *u* [16, thm. 2.3], [27, eqn (18)]. The model (6.2) can be improved accordingly, but this has no impact on the analysis discussed here.

When multiple floating-point operations are performed in a sequence, rounding errors accumulate. For example, if *s* = *x*_1_*y*_1_ + *x*_2_*y*_2_ + *x*_3_*y*_3_ is computed in floating-point arithmetic, the computed $\hat{s}$ satisfies

$$\begin{array}{rl} \hat{s}=\; & (\left(x_{1}y_{1}(1+\delta_{1})+x_{2}y_{2}(1+\delta_{2})\right)(1+\delta_{3})+x_{3}y_{3}(1+\delta_{4}))(1+\delta_{5})\\ =\; & x_{1}y_{1}(1+\delta_{1})(1+\delta_{3})(1+\delta_{5})+x_{2}y_{2}(1+\delta_{2})(1+\delta_{3})(1+\delta_{5})+x_{3}y_{3}(1+\delta_{4})(1+\delta_{5}), \end{array}$$

for some *δ*_1_, …, *δ*_5_ of magnitude at most *u*. It is clear from this example that rounding error analysis for vector and matrix operations involves dealing with multiple terms of the form $\prod_{i=1}^{n}(1+\delta_{i})$. The following lemma [15, Lem. 3.1] bounds the distance between 1 and the product of *n* terms of the form (1 + *δ*_*i*_)^±1^ by means of the quantity

$$\gamma_{n}=\frac{nu}{1-nu},$$

a ubiquitous constant in rounding error analysis.

**Lemma 6.1.**

*If* |*δ*_*i*_| ≤ *u* *and* *ρ*_*i*_ = ±1 *for* *i* = 1, …, *n*, *and* *nu* < 1, *then*

6.3 $$\prod_{i=1}^{n}{\left(1+\delta_{i}\right)}^{\rho_{i}}=1+\theta_{n},\;|\theta_{n}|\leq \gamma_{n}.$$

Under SR, we define the elementary floating-point operations +,−,×,/ to be the stochastically rounded exact ones. Therefore, for SR, (6.2) holds with *u* replaced by 2*u*:

6.4 fl(x op y)=(x op y)(1+δ),|δ|≤2u, op∈{+,−,×,/}.

Standard rounding error analysis based on the model (6.2) clearly remains valid for (6.4), with *u* replaced by 2*u*. This means that one will necessarily obtain larger worst-case error bounds for SR than for RN. However, SR injects randomness into the rounding errors and so we can intuitively expect it to cause the final error in a computation to be smaller than in the worst case, and hence possibly smaller than for RN in some circumstances. In the next subsection, we explain why this intuition is correct.

### 6.1. Probabilistic error analysis

Modelling rounding errors as random variables to obtain probabilistic error bounds is an old idea going back to von Neumann & Goldstine [28], Henrici [29–31] and Hull & Swenson [9], among others. This line of thought has led to the following rule of thumb: a realistic bound on the rounding error of a linear algebra algorithm can be obtained by replacing, in a worst-case error bound, all constants that depend on the dimensions of matrices and vectors by their square roots. The key idea is to exploit the statistical effects of these random variables in the propagation of rounding errors; see, for example, [32, p. 318]. Higham & Mary [33] provided the first rigorous proof of the validity of this criterion: they showed that for random independent zero-mean rounding errors *δ*_*i*_, the constant *γ*_*n*_ in (6.3), can be replaced by

$${\tilde{\gamma }}_{n}(\lambda )=\exp\left(\frac{\lambda \sqrt{n}u+nu^{2}}{1-u}\right)-1=\lambda \sqrt{n}u+O(u^{2}),$$

with a modest constant *λ* > 0 with high probability. Subsequently, Higham & Mary [34] and Ipsen & Zhou [35] obtained a probabilistic error bound for inner products that only requires mean independence of rounding errors, an assumption weaker than independence. Connolly *et al*. [1] derived the following probabilistic version of Lemma 6.1 under these assumptions [1, thm. 4.6]. Here, E[X] denotes the expectation of the random variable *X*.

**Theorem 6.2.**

*Let* *δ*_1_, *δ*_2_, …, *δ*_*n*_ *be random variables of mean zero such that* $E(\delta_{i}|\delta_{i-1},\ldots ,\delta_{1})=E(\delta_{i})=0$, *for* *i* = 2, …, *n*. *If* |*δ*_*i*_| ≤ *u* *and* *ρ*_*i*_ = ±1, *for* *i* = 1, …, *n*, *then for any constant* *λ* > 0,

6.5 $$\prod_{i=1}^{n}{\left(1+\delta_{i}\right)}^{\rho_{i}}=1+\theta_{n},\;|\theta_{n}|\leq {\tilde{\gamma }}_{n}(\lambda )$$

*holds with probability at least* 1 − 2exp ( −*λ*^2^/2).

For RN, the assumptions in theorem 6.2 on the zero mean and mean independence of rounding errors are often satisfied in practice, but it is easy to construct examples where either of the assumptions fails and the backward error almost attains the worst-case bound (6.3) (and thus exceeds the probabilistic bound (6.5) by a factor $\sqrt{n}$).

Importantly, however, SR always satisfies the conditions of theorem 6.2, as shown by the following result [1, lem. 5.2].

**Theorem 6.3.**

*Let the computation of interest generate rounding errors* *δ*_1_, *δ*_2_, …, *in that order. If SR is used then the* *δ*_*i*_ *are random variables of mean zero such that* $E(\delta_{i}|\delta_{i-1},\ldots ,\delta_{1})=E(\delta_{i})(=0).$

It follows that the probabilistic bound (6.5) holds unconditionally for SR provided that is *u* replaced by 2*u* in view of (6.4). Hence for SR, the rule of thumb that one can replace *nu* in a worst-case error bound by $\sqrt{n}u$ is a *rule*.

The probabilistic bound is most favourable when the worst-case bound is approximately attained. One situation in which this happens is when many tiny increments are applied to a relatively large quantity. If *ϕ* ∈ *F* is updated by increments *h*_1_, *h*_2_, …, which have magnitude smaller than half of the spacing of the floating-point numbers around *ϕ*, then using RN gives *ϕ* = fl(*ϕ* + *h*_1_) = fl(fl(*ϕ* + *h*_1_) + *h*_2_) = ···, and the information in the updates is lost. This phenomenon, known as *stagnation*, commonly occurs in practical applications. It arises, for example, in neural networks, when parameter updates become very small, or in numerical methods for ODEs and partial differential equations (PDEs), when a very small time step is chosen. SR avoids stagnation, as some of the updates produce rounding that changes the partial sum. This can be seen from the following result [1, thm. 6.2].

**Theorem 6.4 (inner products).**

*Let y* = *a*^*T*^*b*, *where* $a,b\in R^{n}$, *be evaluated in floating-point arithmetic. Under SR, the computed* $\hat{y}$ *satisfies* $E(\hat{y})=y$ *regardless of the order in which the sums of products are evaluated*.

Taking *b*_*i*_ ≡ 1 in the theorem, we see that the expected value of a sum is the true value under SR. As a simple example, suppose we run the code

*x* = 1;
**for** *i* = 1 : 10 **do**
 *x* = *x* + *ɛ*_*M*_/4
**end**

in floating-point arithmetic. Since the spacing of the floating-point numbers between 1 and 2 is *ɛ*_*M*_ = 2*u*, with RN every addition rounds down and the computed result is $\hat{x}=1$. With SR, however, each addition has a probability 1/4 of rounding up, giving an increment of *ɛ*_*M*_. Hence the expected result is 1 + 10 · (1/4) · *ɛ*_*M*_, which is the exact result, albeit not a floating-point number in the working precision.

We emphasize that the benefits of SR are not restricted to curing stagnation. This rounding mode ensures zero-mean rounding errors, so can produce smaller errors than RN in situations where RN systematically produces rounding errors of one sign (see [33, §4.2.2] for an example).

## 7. Implementation

Here, we discuss how to implement SR.

### 7.1. SR expressed in terms of other rounding modes

We can express the SR operator in terms of other rounding operators by writing, for x∉F,

7.1 $$\mathrm{fl}(x)=\left\{ \begin{array}{rl} \text{RA}(x),\; & \text{with probability}\;q(x),\;\\ \text{RZ}(x),\; & \text{with probability}\;1-q(x), \end{array}\right.$$

where RA denotes the operator that rounds away from zero and *q*(*x*) ∈ [0, 1]. For mode 1 SR, we can rewrite (2.2) as

7.2 $$q(x)=\frac{x-\text{RZ}(x)}{\text{RA}(x)-\text{RZ}(x)}.$$

In order to implement (2.1) or (7.1) in practice, we need to define a discrete version of the SR operator. Given a positive integer *k*, which controls the number of bits used to approximate the continuous definition (2.1), let *P* be a random precision-*k* floating-point number drawn from the uniform distribution over the interval [0, 1).^2^ We have that, for x∉F,

7.3 $$\mathrm{fl}(x)=\left\{ \begin{array}{cc} \text{RA}(x),\; & \text{if}\;P<\frac{x-\text{RZ}(x)}{\text{RA}(x)-\text{RZ}(x)},\;\\ \text{RZ}(x),\; & \text{if}\;P\geq \frac{x-\text{RZ}(x)}{\text{RA}(x)-\text{RZ}(x)},\; \end{array}\right.$$

where SR, RA, RZ round to some precision *p*. It is worth noting that the choice of the optimal *k* for implementing SR is one of the main open questions surrounding mode 1 SR. A lower value of *k* makes a hardware implementation cheaper but is expected to reduce the accuracy benefit that SR may potentially bring: setting *k* = 1, for example, gives mode 2 SR.

While (7.3) is an accessible definition of SR, most of the implementations discussed in this section do not take the comparison-based approach that (7.3) would suggest. On the contrary, they add bits from a random stream to the part of the number that will be truncated. The equivalence between this is the idea, which we will discuss in much greater detail in §§7.3 and 7.4, and (7.3) is shown in [37, §4].

Table 3 compares the features of SR in a number of implementations available.

**Table 3.** Summary of SR implementations. Here, *p* is the target precision of rounding; *k* is the precision of the random number used for SR; the ‘type’ column has ‘I’ for integer or fixed-point arithmetic and ‘F’ for floating-point arithmetic; the ‘op.’ column indicates the class of operations supported or ‘any’ if rounding of any operation is supported; the ‘H/S’ column has H if SR is in hardware and S if software; and in the ‘applications’ column ML and QC stand for machine learning and quantum computing, respectively.

reference	type	*p*	*k*	op.	H/S	applications
Barnes *et al.* (1951) [5]	I	8	1	×, /	H	general
Gupta *et al.* (2015) [7]	I	18	30	dot prod.	H	ML
Davies *et al.* (2018) [38]	—	7	—	×, +	H	ML
Higham & Pranesh (2019) [2]	F	≤64	64	any	S	general
Hopkins *et al.* (2020) [39]	I	32	2–32	×	S	ODE solve
Mikaitis (2020) ([8,40]	I, F	≤32	32	any	H	general
Meurant (2020) [41]	I, F	≤64	64	any	S	general
Fasi & Mikaitis (2020) [42]	F	≤64	64	any	S	general
Croci & Giles (2020) [43]	F	≤32	32–64	+, × , /	S	general/PDEs
Fasi & Mikaitis (2021) [37]	F	*p*	*p*	+, $\times ,/,\sqrt{}$	S	general
Paxton *et al.* (2021) [44]	F	≤32	32–64	any	S	general
Klöwer (2021) [45]	F	≤32	32–64	any	S	general
Krishnakumar & Zeng (2021) [46]	I	*n*	*m* = *n*	×	—	QC

https://github.com/milankl/StochasticRounding.jl

### 7.2. Proposed IEEE 754 style properties of SR

The definition in the previous section does not cover edge cases such as overflow, underflow and rounding of infinities and NaNs (not-a-number). In the following, we propose our definition of SR for these edge cases by giving some properties of SR analogous to those of the rounding modes defined in the IEEE 754 standard [12].

— If the exact number is in the range of the target format, SR should be performed as though the number was originally held in *p* + *k* bits and then rounded to *p* bits according to (2.1). Here, the extra *k* bits refer to the *precision of SR*, as well as the number of random bits required.
— Overflows: if the exact number lies between the maximum representable number ±*f*_max_ and the neighbouring value that is not representable in the target format and will be treated as ±∞, SR is performed as though the value is representable, to preserve the statistical information about the round-off bits.
— When the exact number is smaller than the smallest value representable in the target format, SR should round stochastically to one of the two neighbouring floating-point values in the target format, either zero or the smallest representable value, maintaining the sign.
— When subnormals are disabled or not supported in the target format and the exact value is in the range of underflow, SR should round either to zero or to the smallest normalized value, again without changing the sign.
— ±∞ and ±0 should not be rounded (changed) by the SR operation. NaNs with payloads that cannot be represented in the target format should not be stochastically rounded: a NaN with an implementation-defined payload may be returned, as per IEEE 754 [12, §6.2.3] (relevant in a mixed-precision setting, for example converting binary64 to binary32).
— As in the standard rounding operations [12, §4.3], inexact, underflow and overflow exceptions should be signalled by the SR operation.

### 7.3. Modifying basic floating-point algorithms to include SR

Now we discuss how to modify classical algorithms for addition and multiplication of floating-point numbers [16, ch. 7], [27, §4.2.1], [47, ch. 8] in order to obtain algorithms that support SR and can readily be implemented in software or hardware. Algorithms for other operations to include SR such as fused multiply-add (FMA) or division can be similarly derived by modifying the original algorithms for the IEEE 754 arithmetic operations [16,47]. To the best of our knowledge, these algorithms are new in that no general methods to round to precision *p* taking normalization into account had so far been proposed in the literature.

**Addition.** The sum *r* = \circ(*x* + *y*), where \circ ∈ {RN, RZ, RD, RU} and *x* and *y* are binary floating-point numbers, can be computed as we now explain. Let $x={\left(-1\right)}^{s_{x}}\times m_{x}\times 2^{e_{x}-p+1}$ and $y={\left(-1\right)}^{s_{y}}\times m_{y}\times 2^{e_{y}-p+1}$ be two normalized precision-*p* floating-point numbers. We assume that *s*_*x*_ = *s*_*y*_ = 0, which implies that *x* and *y* are positive, in order to avoid considering sign interactions which may transform the addition into a subtraction. This restriction does not affect our main observations pertaining to the implementation of SR. The role that the sign of the operands plays in this algorithm is discussed, for instance, in [16, §7.3]. We make additional observations about subtraction when necessary.

The sum $r=\circ (x+y)={\left(-1\right)}^{s_{r}}\times m_{r}\times 2^{e_{r}-p+1}$ is computed as follows:

(i) If *e*_*y*_ > *e*_*x*_, swap *x* and *y* to ensure that *e*_*x*_ ≥ *e*_*y*_.
(ii) Alignment of the significands: compute $m_{y}\times 2^{-(e_{x}-e_{y})}$ by shifting *m*_*y*_ to the right by *e*_*x*_ − *e*_*y*_ places. Set *e*_*r*_ = *e*_*x*_. It is not necessary to keep all the bits that are shifted out: maintaining only two bits plus a third *sticky bit* suffices—see below.
(iii) Sum of the significands: compute $m_{t}=m_{x}+m_{y}\times 2^{-(e_{x}-e_{y})}$. At this step, *m*_*t*_ is an exact sum of the significands.
(iv) Normalization of the result: since 0 ≤ *m*_*t*_ < 2^*p*+1^, we may need to normalize the result by shifting *m*_*t*_ to the right by one place (if *m*_*t*_ ≥ 2^*p*^) and increasing *e*_*r*_ by 1 (note that a shift left may be needed with subtraction—see below).
(v) Rounding: the significand of the rounded sum, *m*_*r*_, is computed by rounding the normalized exact sum *m*_*t*_ to *p* significant bits according to ∘, and renormalizing if required. At this point, *r* is the correctly rounded sum of *x* and *y*.

In order to perform the rounding at step (v) of the algorithm, it may seem necessary to preserve all the bits that, being after the bit in the *p*th position, are dropped off during steps (ii) and (iv). It can be shown, however, that for \circ ∈ {RN, RZ, RD, RU} it suffices to keep only the first two discarded bits after the one in position *p* plus an extra specially computed bit. These are the *guard bit* G, in position *p* + 1, the *round bit* R, in position *p* + 2, and the *sticky bit* T, in position *p* + 3, which is a logical OR of all the bits after the (*p* + 2)nd. Together, these three bits are called in short the GRT bits [47, §8.4.3]. In the algorithm above, they are formed in step (ii) and updated in step (iv) if normalization is required.

**Figure 2.**
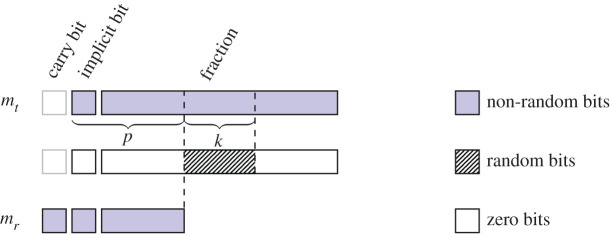
Alignment of bits in algorithms for stochastic rounding based on sums. The random bits are added to the significand *m*_*t*_ followed by the truncation of it. How the bits are generated and added depends on the implementation—we may only add the *k* bits to the top *k* bits of the bottom part of the significand and then use a carry-out bit to control the rounding of *m*_*r*_ after the truncation, or we may pack the *k* random bits in a word as long as *m*_*t*_ and add it to *m*_*t*_ using integer arithmetic: the propagating carry will cause rounding in the top *p* bits.

We now explain how the algorithm should be modified in order to include SR as an option in step (v). Figure 2 demonstrates stochatic rounding of *m*_*t*_ pictorially. We use the same notation as in §7.1, and use *k* to denote the number of bits used for rounding, or equivalently the number of bits in the random number used to perform the rounding. In step (ii), instead of computing the GRT bits from the shifted-out bits of *m*_*y*_, we keep the *k* trailing bits beyond that in position *p*. Depending on the implementation, it might be necessary to manipulate appropriately those extra bits when subtraction is considered. An alignment of more than *p* + *k* − 1 bits is unnecessary, as in that case SR with *k* bits would not have any effect and the largest summand *x* would be returned unchanged. If a shift is required in step (iv), the whole *p* + *k*-bit significand has to be shifted in order to keep the trailing *k* bits correct after the normalization. In order to perform step (v), it is necessary to generate *k* bits from a stream of uniformly distributed random bits. This operation is expensive, but can be performed asynchronously at any point before reaching the last step, as it does not require any information about *x* or *y*. Finally, the *k* bits from the random stream are added to the *k* bits immediately following the first *p* bits of the normalized *m*_*t*_; if this operation leads to a carry-out, we increment the top *p* bits of *m*_*t*_ by 1 and truncate the bits after the first *p* bits to form the rounded significand *m*_*r*_. Implementing SR by adding random bits to the fraction is almost universally used in the software and hardware implementations discussed in §7.4 and §7.5 below.

We need to consider normalization and whether the *k* bits required for rounding could be altered by shifting. Shifting is necessary in three cases. First, when the addition of the significands causes a carry-out, *m*_*t*_ is normalized by means of a one-place shift to the right, which does not violate the bottom *k* bits. Secondly, when the difference of exponents *e*_*x*_ − *e*_*y*_ is larger than 1, the smaller operand is aligned so that there are multiple zeros at the front and consequently only a left shift by one position may be required on effective subtraction. For this reason, one extra bit is needed to make sure that a 1 that drops off the *p* + *k* bits is shifted in correctly by the left shift. Thirdly, when the exponent difference is 1 cancellation may occur on effective subtraction and multiple left shifts may be required to normalize the result. Since the alignment was performed by shifting right by only one place; however, there is no risk of any bits being shifted beyond the (*p* + *k*)th position, and therefore no incorrect bits will be shifted in during the normalization. Therefore, only one extra bit is necessary, and the width of *m*_*t*_ should be *p* + *k* + 1 bits.

If the sum of two floating-point numbers is subnormal, then it is exact and no rounding is required [16, thm. 4.2]. If one of the inputs is subnormal, then the significand alignment step requires minor modifications as per [16, §7.3.3], while the rest of the algorithm for the floating-point addition with SR remains the same.

Addition of floating-point numbers can result in the following exceptions: overflow, underflow, inexact and NaN [47, p. 425]. With SR these may be handled as discussed in §7.2.

*Multiplication.* Given two normalized positive floating-point numbers *x* and *y* as in the previous section, the product *r* = \circ (*x* × *y*) can be computed as follows:

(i) Product of the significands: compute the 2*p*-bit integer *m*_*t*_ = *m*_*x*_ × *m*_*y*_. The fact that 2^*p*−1^ ≤ *m*_*x*_, *m*_*y*_ < 2^*p*^ implies that 2^2*p*−2^ ≤ *m*_*t*_ < 2^2*p*^.
(ii) Sum of the exponents: compute *e*_*r*_ = *e*_*x*_ + *e*_*y*_. At this point, we have the exact product $m_{t}\times 2^{e_{r}-2p+2}$.
(iii) Normalization of the result: if *m*_*t*_ ≥ 2^2*p*−1^ we need to normalize the result by shifting the significand by one place to the right and increasing *e*_*r*_ by 1.
(iv) Rounding: the normalized exact product *m*_*t*_ is rounded to *p* significant bits according to ∘, giving *m*_*r*_. At this point, *m*_*r*_ is the rounded product of *x* and *y*.

As with addition, the G and T bits are required to perform the rounding in step (iv) (the R bit is not required since left shifts cannot occur here). After the normalization in step (iii), the unrounded result will be in the top *p* bits, whereas the bottom *p* bits will be used for rounding. In order to implement SR with *k* random bits (in this case *k* ≤ *p*, as *m*_*t*_ has at most 2*p* bits and there is no need to consider larger values of *k*) we need to add a *k*-bit random number to the top *k* of the bottom *p* bits of the internal significand *m*_*t*_: a carry will increment the *p*th bit of the top segment of *m*_*t*_, causing the number to round up. The significand of the stochastically rounded result will consist of the top *p* bits of *m*_*t*_.

For subnormal values, a few modifications have to be incorporated in the multiplication algorithm. First of all, if both inputs are subnormal, then the product will be in the underflow range and SR can be handled as in §7.2. If the inputs are normalized but yield a subnormal result, it is necessary to shift *m*_*t*_ right by more than one place in step (iii), depending on how much the product’s exponent differs from that of subnormals in the target format. If only one of the inputs is subnormal, then the product can be either normal or subnormal. Two approaches are taken in this case: either the subnormal input is normalized before the multiplication, or the product’s significand is normalized by a left shift. We do not go into details, as this does not change the SR algorithm (SR is performed in step (iv) after the product has been normalized or denormalized as required), and refer the reader to [16, §7.4.2].

Multiplication of floating-point numbers can result in the following exceptions: overflow, underflow, inexact and NaN [47, p. 438]. With SR these may be handled as discussed in §7.2.

### 7.4. Simulation of SR in software

SR can be simulated in software in a straightforward fashion by relying on high-precision floating-point arithmetic. The computation is performed in higher precision and the high-precision result is rounded using (7.1), where *q*(*x*) in (7.2) is based on the (higher-precision) approximation to *x* rather than on its exact value. This approach is easy to implement as long as higher-than-working-precision arithmetic is available, be it in hardware, for instance when emulating binary32 rounding using binary64 arithmetic, or in software, through arbitrary precision libraries such as the GNU Multiprecision Library (GNU MPFR) [48].

In practice, once the high-precision solution has been computed, the rounding step can be performed in several ways. The Matlab function chop^3^ [2] and the FLOATP_Toolbox^4^ for Matlab [41] leverage the Matlab random number generator to draw a random number *r* from the uniform distribution over the open interval (0, 1) and choose the rounding direction depending on whether *r* is larger or smaller than *q*(*x*). These software packages directly implement SR through floating-point comparison operations as in (7.3).

Most software favours the use of integer random numbers, integer arithmetic and bit manipulation.

**Figure 3.**
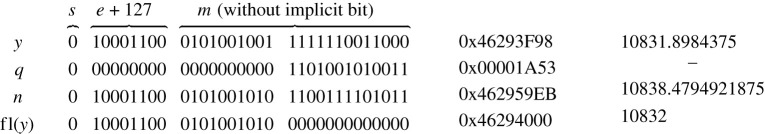
Example: rounding a binary32 number *y* to a format with *p* = 11 significant digits (including the implicit bit) using the SR algorithm implemented in QPyTorch. The number *n* is the integer sum of the two bit strings representing *y* and *m*; fl(*y*) is obtained by zeroing out the trailing 24 − *p* = 13 trailing bits of *n*. For floating-point numbers, the three binary strings represent the sign (*s*), the unbiased exponent (*e*) and the integer significand without the implicit bit (*m*); the last group is further divided into a group of *p* − 1 bits to keep and 24 − *p* bits to zero out. We also report the hexadecimal string representing the numbers, and for floating-point numbers the corresponding exact decimal representations.

The implementation of SR in the QPyTorch^5^ package [49], for example, rounds stochastically a binary32 number *y* to a floating-point format with precision *p* < 23 as follows. First, it generates a 32-bit integer *q* by zeroing out the leading (*p* − 1) + 9 bits of a 32-bit random integer. These zeros are introduced in positions that correspond to the sign of *y* (1 bit), its exponent (the next 8 bits), and the *p* most significant bits of its significand (the following *p* − 1 bits, in view of the implicit bit conversion). Next, the algorithm computes $n=\tilde{y}+q$, where $\tilde{y}$ is the binary representation of *y* seen as an integer, uses a bitmask to zero out the 24 − *p* trailing digits of *n*, as 24 is the number of precision bits in a binary32 number, and finally returns the value thus obtained as a 32-bit floating-point number. An example illustrating this rounding technique is provided in figure 3. The implementations in the CPFloat^6^ C library [42] use an analogous technique when rounding binary32 as well as binary64 floating-point numbers to lower precision. The same approach is followed by Verificarlo^7^ [50], an instrumentation tool which uses the GNU Compiler Collection (GCC) quad format as extended precision for binary64, and binary64 as extended precision for binary32.

The approach of simulating SR through extended precision is also used in the mcaquad back-end of the Valgrind tool Verrou^8^ [51,52]. Verrou offers a second back-end, called verrou, which performs SR without using higher precision. The algorithms in the verrou back-end use double-double arithmetic, and emulate high-precision computations implicitly by representing numbers with at least 106 bits of precision as the unevaluated sum of two binary64 values. These techniques are based on reduction operations [12, §9.4], also known as error-free transformations, and are used to approximate the distance between the exact result of a computation and the two rounding candidates in binary64 arithmetic. These are then used to approximate the rounding probabilities.

Fasi & Mikaitis [37] propose a similar but more general approach for implementing in software stochastically rounded elementary arithmetic operations: addition/subtraction, multiplication, division and extraction of the square root. For each operation, they propose two algorithms, one that uses only RN, and one that combines it with RZ, RU and RD. These algorithms compute first the result of the elementary arithmetic operation and then the error induced by the rounding. The rounding direction is chosen by adding a pre-generated random number to the computed error, and then adding this quantity to the result of the operation—Fasi & Mikaitis [37] show that this procedure is equivalent to the direct implementation of SR via (7.3), which relies on a comparison-based approach. In numerical experiments, both types of algorithms are faster than a C implementation that relies on the GNU MPFR library, and the RN-only versions are faster on x86 architectures, where switching the rounding mode incurs a high performance penalty [16, §12.3.2].

Klöwer’s Julia software package StochasticRounding.jl^9^ defines three new Julia floating-point types that automatically include SR. These correspond to bfloat16, binary16 and binary32 (table 1), and use the Xoroshiro128Plus fast pseudo-random number generator (PRNG).^10^ Composability and type flexibility in Julia enable SR computations in single and half precision in a large number of numerical software and mathematical libraries. Automatic application of SR operations is extremely advantageous from a user standpoint, as it allows significant code simplification. To implement (7.3), the implementation is also based on the approach of adding random bits to the part of the floating-point number that will be truncated.

In terms of fixed-point arithmetic with SR, Hopkins *et al.* [39] and Mikaitis [40] have recently implemented a set of rounding and multiplication operations^11^ and used them on low-power ARM integer processors. Multiplication routines for various fixed-point formats in the ISO 18037 embedded C standard [53] were developed by exploiting the fact that ARM processors return the full-precision result of integer multiplication using two registers: multiplying two 32-bit fixed-point values, for example, returns the exact 64-bit result with all the information of integer and fraction bits of products preserved. The bottom bits of the fraction can then be used to round the results to one of the standard fixed-point formats stochastically. In this implementation, the comparisons between the random numbers and the round-off bits are implemented directly as in (7.3), except using integer arithmetic.

### 7.5. Overview of available devices and patents

Now we review hardware designs discussed in the literature, some of which are already available in commercial hardware.

The Graphcore Intelligence Processing Unit (IPU) is a highly parallel machine learning accelerator that supports SR for binary32 and binary16 arithmetic [54, §2.1], [55, ch. 10], [56] (table 1). The patent filed by Graphcore [57] reveals some technical details that are not specified in the documentation but may reflect the hardware implementation of the IPU. The document explains how binary32 values are stochastically rounded to binary16 precision in hardware by using a PRNG, also implemented in hardware—this kind of conversion might be performed in the IPU, although this is not reported. The algorithm begins by generating a 24-bit random number, that is, one random bit for each bit in the significand of a binary32 value. It then uses 13 or more of those bits to round a number to binary16. The number of random bits that are actually used depends on whether rounding will result in a normal or subnormal number. The actual rounding is performed by adding these random bits to the part of the significand that will be discarded. This operation may generate a carry bit to be added to the least significant bit of the significand of the input truncated to 11 significant bits. A non-zero carry bit will cause the absolute value of the number to round up. This type of implementation is used in most of the references mentioned below.

Two patents filed by IBM disclose methods for implementing floating-point adders [58] and multipliers [59] that use SR. The authors demonstrate the techniques on an 8-bit data type, but mention binary32 and binary64 as examples of other formats to which the approach can be applied. The procedures require a fixed number of random bits be loaded into a register, but the patents do not explicitly mention how these bits should be generated. SR is performed by using the bits that drop off when fitting the result of the adder or multiplier into the 8-bit format, and as in the case of the Graphcore IPU, the rounding step is implemented by adding the random bit stream to the round-off bits, in both the adder [58] and the multiplier [59]. It is not mentioned, however, whether the sum is normalized before being rounded, and if so whether the bits that are to be shifted out during the normalization are also taken into account when rounding—in the algorithms outlined in §7.3 we propose that the rounding step should be performed after the normalization for a correct implementation.

A patent from AMD describes methods and circuits to use SR in conjunction with integer adders or accumulators [60]. The document shows the design of (i) a mixed-precision adder that computes the sum of a 32-bit and a 16-bit number by using a 16-bit random number supplied through a third input lane, and (ii) a 32-bit accumulator which takes as inputs both the next 16-bit value to accumulate and a 16-bit random number, and returns a 16-bit stochastically rounded sum—once again, SR is implemented by adding random bits to the round-off digits, as was the case for the Graphcore IPU. The pseudo-random numbers in the proposed SR unit are generated with a *linear-feedback shift register* (LFSR), but no specific algorithm is mentioned.

A patent from NVIDIA demonstrates a method to round stochastically floating-point values to lower precision, using a fixed, programmable, or computable rounding bit position [61]. The authors explain how to round binary64 values to binary32, and binary32 values to binary16 and bfloat16. A distinctive feature of this design is that it performs SR by using the bottom bits of the significand of the number to be rounded without relying on a random number generator [61, fig. 2B]. For example, the 23-bit fraction of a binary32 number can be rounded to the 10-bit fraction of a binary16 value by setting *k* = 8, taking the bottom 8 bits of the fraction in place of a random bit stream, aligning and adding them to the significand as in figure 2, and finally setting the 13 least significant bits to zero. The authors note that this method for performing SR has an advantage over using real random numbers, since it is deterministic and cheaper to implement. They do not mention, however, whether replacing the random number with part of the input causes SR to lose any of its desirable properties.

Gupta *et al.* [7] discuss the hardware prototype of a fixed-point matrix multiplier based on a two-dimensional (2D) *systolic array architecture* and demonstrate experimental results from a *field-programmable gate array* (FPGA) implementation. Each node of the systolic array is a *multiply-and-accumulate digital signal processing* (MACC DSP) unit that multiplies two integers and accumulates the result into an internal register. Each element of the matrix product is produced by a single MACC DSP unit. The hardware is generalized, but the authors report results for an implementation in which each MACC DSP unit accepts inputs of at most 18 bits and accumulates the partial results in an internal 48-bit register. When the matrix product is computed, each 48-bit element is passed through an SR unit (there is one for each column of the 2D array of MACC DSP units) to produce the 18-bit rounded and saturated results. The pseudo-random numbers needed to implement SR are generated using an LFSR. The 30 random bits are added to the 30 least significant bits of the 48-bit internal register. This may cause a carry to propagate to the 18 most significant bits of the result. Finally, the trailing 30 bits are set to zero, thereby producing the rounded number.

The Intel Loihi [38] and the SpiNNaker2 [8,40,62] digital *neuromorphic* processors include SR. The Intel Loihi processor has multiply-accumulate hardware that computes a 7-bit approximation to *x*[*t*] = *α* · *x*[*t* − 1] + *δ* · *s*[*t*] (with *s*[*t*] ∈ {0, 1}, *α* a decay factor, and *δ* an impulse amount added at each step). It is not specified where SR is applied in this computation, what precision and type of random numbers are used, and how SR is implemented. The SpiNNaker2 SR accelerator rounds and saturates 64-, 32- or 16-bit to 32- or 16-bit fixed-point numbers with SR. As a special case, it also includes rounding from IEEE 754 binary32 to bfloat16. The random bits needed for rounding are produced using the 32-bit hardware pseudo-random number generator available on SpiNNaker2 [62]. The number of bits to be used for rounding is programmable for fixed-point formats (it can be anything between 1 and 32 bottom bits of the input) and is fixed to 16 for binary32-to-bfloat16 rounding. As in the implementations mentioned above, SR is performed by adding the random bits to the round-off bits (figure 2).

## 8. Applications

In applications, SR can replace existing rounding modes (usually RN) either globally or in certain parts of an algorithm, and either true random numbers or pseudo-random numbers can be used. The latter are often preferred as they ensure reproducibility of the result. In this section, we review applications where SR has been applied, and in some cases provide Matlab experiments for demonstration, which are made available on GitHub.^12^

### 8.1. Numerical linear algebra

For most numerical linear algebra algorithms, rounding error analysis builds on Lemma 6.1, or some variation of it, thus these algorithms can benefit from the smaller bound guaranteed for SR by the probabilistic error analysis of Theorem 6.2. For inner products, in particular, we have the following result [1, thm. 4.8].

**Theorem 8.1 (inner products).**

*Let* *y* = *a*^*T*^*b*, *where* $a,b\in R^{n}$, *be evaluated in floating-point arithmetic with SR. Then for any constant* *λ* > 0 *the computed* $\hat{y}$ *satisfies*

8.1 $$\hat{y}={\left(a+\Delta a\right)}^{T}b=a^{T}(b+\Delta b),\;|\Delta a|\leq {\tilde{\gamma }}_{n}(\lambda )|a|,\;|\Delta b|\leq {\tilde{\gamma }}_{n}(\lambda )|b|$$

*with probability at least* 1 − 2*n*exp ( − *λ*^2^/2) *regardless of the order of evaluation*.

The worst-case error bound corresponding to (8.1) has the same form but with the constant ${\tilde{\gamma }}_{n}(\lambda )$ replaced by *γ*_*n*_, which is roughly a factor $\sqrt{n}$ larger [15, §3.1].

As a special case, we can take *b*_*i*_ ≡ 1 and deduce that

$$|\sum_{i=1}^{n}a_{i}-\mathrm{fl}\left(\sum_{i=1}^{n}a_{i}\right)|\leq {\tilde{\gamma }}_{n}\sum_{i=1}^{n}|a_{i}|.$$

**Figure 4.**
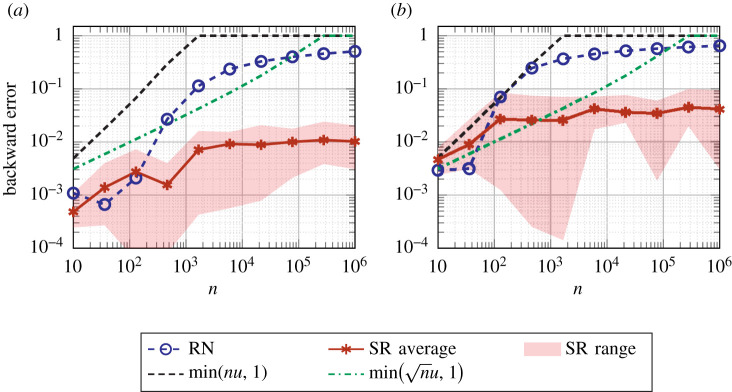
Relative errors for computing $\sum_{i=1}^{n}1/i$ with RN and SR. The densely dashed and dash-dotted lines are the worst-case error bound for RN and the probabilistic error bound for SR (with *λ* = 1), respectively. Stochastic rounding experiments are repeated 10 times; the solid line represents the average error, the edges of the shaded area the minimum and maximum error. (*a*) binary16 arithmetic, (*b*) bfloat16 arithmetic.

Figure 4 plots the relative errors for the sum $\sum_{i=1}^{n}\mathrm{fl}(1/i)$ computed in binary16 (table 1) with RN and SR for a range of *n*. Note that the summands are already converted to binary16 (with RN), so the only errors are in the summation. This example models a very slowly growing sum of decaying summands. We see that SR has much smaller errors than RN for larger *n* and that the errors for SR are mostly well within the probabilistic bound with *λ* = 1.

Matrix products are considered in the following result [1, thm. 4.9], in which we denote by *a*_*j*_ the *j*th column of a matrix *A*.

**Theorem 8.2 (matrix–matrix products).**

*Let* *C* = *AB* *with* $A\in R^{m\times n}$ *and* $B\in R^{n\times p}$ *be evaluated in floating-point arithmetic with SR. For any* *λ* > 0, *the* *jth column of the computed* $\hat{C}$ *satisfies*

8.2 $${\hat{c}}_{j}=(A+\Delta A_{j})b_{j},\;|\Delta A_{j}|\leq {\tilde{\gamma }}_{n}(\lambda )|A|,\;j=1,\ldots ,n,$$

*with probability at least* 1 − 2*mn*exp ( − *λ*^2^/2), *and hence*

8.3 $$|C-\hat{C}|\leq {\tilde{\gamma }}_{n}(\lambda )|A||B|,$$

*with probability at least* 1 − 2*mnp*exp ( −*λ*^2^/2).

The worst-case error bounds corresponding to (8.2) and (8.3) have the same form but with $\tilde{\gamma }(\lambda )$ replaced by *γ*_*n*_ [15, §3.5].

**Figure 5.**
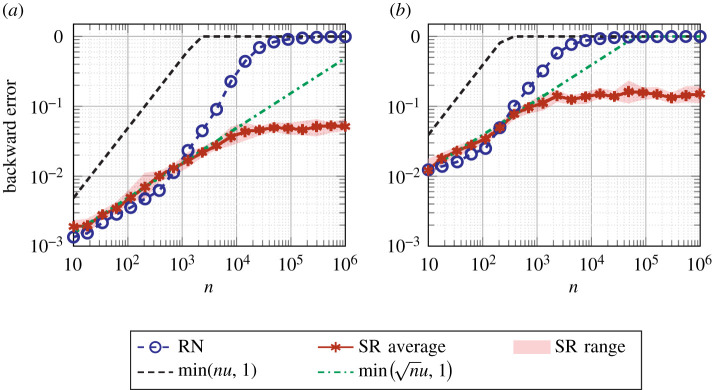
Backward error for computing *y* = *Ax* with RN and SR, where $A\in R^{100\times n}$ has entries drawn from the uniform distribution over [0, 10^−3^] and $x\in R^{n}$ has entries sampled from the uniform distribution over [0, 1]. The densely dashed and dash-dotted lines are the worst-case error bound for RN and the probabilistic error bound for SR (with *λ* = 1), respectively. Stochastic rounding experiments are repeated 10 times; the solid line represents the average error, and the edges of the shaded area the minimum and maximum error. (*a*) binary16 arithmetic, (*b*) bfloat16 arithmetic.

This result is illustrated in figure 5, which plots the backward error for computing a matrix–vector product *y* = *Ax* where $A\in R^{100\times n}$ has entries drawn from the uniform distribution over [0, 10^−3^] and $x\in R^{n}$ has entries sampled from the uniform distribution over and [0, 1]. We see that RN attains its worst-case rate of error growth and hits a relative error of 1, whereas SR has slower error growth and maintains some accuracy for all *n*. In fact, stagnation (see §6.1) occurs in this example when $n≳10^{3}$ for binary16 and when $n≳10^{2}$ for bfloat16, as shown by the increased rate of error growth from these points onwards for RN.

As this rounding error analysis and the examples illustrate, SR is especially useful for large-scale and/or low-precision computations.

### 8.2. Machine learning

The use of SR in neural networks is not a new idea. Höhfeld & Fahlman [63,64] proposed it in 1992, calling it probabilistic rounding. Today, SR is being used in machine learning in conjunction with half precision arithmetic, not least because of its ability to avoid the problem of stagnation that affects RN. Gupta *et al.* [7] show that SR can be used for training deep neural networks in 16-bit fixed-point arithmetic with little or no degradation in the classification accuracy. Su *et al.* [65] successfully train neural networks in 8-bit fixed-point arithmetic using SR and offer some suggestions as to why SR is beneficial in this context. Muller & Indiveri [66] use SR (called *randomized rounding* or *online stochastic*) to map continuous neural network weights to a discrete low-precision fixed-point representation. It is shown that with SR the networks can perform well with lower precision weights than required with the standard rounding.

Essam *et al.* [67] combine SR with dynamic precision fixed-point arithmetic formats (variable integer scaling factors) in training neural networks. Na *et al*. [68] implement SR with dynamic fixed-point arithmetic in hardware for neural network applications. Used to train neural networks with 24-bit fixed-point numbers, SR provides performance similar to that of binary32, but with lower hardware area and energy costs. The authors also show that without SR even 64-bit arithmetic is not enough to train the kinds of neural networks they used.

Joardar *et al.* [69] implement a 32- to 16-bit fixed-point arithmetic SR unit in an in-memory computing device for neural network training, based on *resistive random access memory* (ReRAM), which uses an LFSR 16-bit pseudo-random number generator. They report that SR added negligible ReRAM cell area overhead with each SR circuit adding less than 1%. SR was implemented by adding a 16-bit pseudo-random number to the bottom 16 bits of a 32-bit addition result and then truncating the result to 16 bits—the same technique as used in most of the hardware designs mentioned in §7.

SR has also been applied in machine learning with floating-point arithmetic. Wang *et al.* [70] train neural networks in 8-bit floating-point arithmetic with SR, obtaining factor 2–4 speed-ups over 32-bit training. Zamirai *et al.* [71] find that either of SR and compensated summation [15, §4.3] enables training in bfloat16 to match 32-bit training. Xia *et al.* [72] use mode 2 SR and show faster convergence in training using 16-bit fixed-point numbers. They modify mode 2 SR, in that instead of rounding only the values that are not representable, all values are rounded, including those that are exactly representable in the current floating-point format. Mellempudi *et al.* [73] show that training neural networks using SR with 8-bit floating-point numbers yields performance comparable to that of binary32. Ortiz *et al.* [74] compare 12-bit fixed- and floating-point formats with and without SR with binary32 arithmetic in the training of neural networks. They find that SR can be very useful in improving the accuracy: in their experiments with a 12-bit fixed-point format, using RN produced a training accuracy of just 10%, while switching to SR produced a training accuracy on par with that of binary32 arithmetic [74, table 2].

Zhang *et al*. [75] use SR with the block floating-point (BFP) [76, p. 26] number representation technique in a training system for deep neural networks. Their system exploits BFP of variable precision over the whole training process, based on the number of training iterations and neural network layers used. In this work, SR is applied in conversion of floating-point values to a lower precision BFP representation [75, §III]. There is some indication [75, fig. 4] that SR is implemented, similarly to most implementations reviewed here, by adding random bit streams to parts of numbers later truncated (as shown in figure 2). A hardware implementation of the proposed training method is also presented, with a matrix multiplier in BFP with SR [75, §V]. To perform SR in hardware, the authors use 8-bit random binary streams from an LFSR which they sum with the significands of BFP numbers [75, §V-C], followed by truncation of the bottom bits. The authors report a 2-6× speed-up over prior approaches when using variable precision BFP for this application. At the same time, they report having similar accuracy to previous approaches, which is attributed to the combination of variable precision BFP and SR.

We note that the survey by Wang *et al.* [77] of custom hardware for deep learning includes a review of work that uses SR. Another survey by Lee *et al.* [78] reviews arithmetic- and implementation-level techniques in deep learning and includes various works that use SR.

There are many more examples of SR being of use in low-precision machine learning applications. For more details, see the references cited in the papers discussed above.

### 8.3. Numerical verification software

Numerical verification software uses SR to explore the propagation of rounding errors in applications: a particular computation is repeated multiple times and the distribution of errors from these runs is used to draw conclusions about the sensitivity of a code to rounding errors. Mode 2 SR, known as stochastic arithmetic [79], is used for example in the CADNA library [80].

An approach that includes as options both mode 1 and mode 2 SR is Monte Carlo arithmetic [81,82], a method used by tools such as Verificarlo [50] and Verrou [51,52]. Monte Carlo arithmetic is more general than SR, not least because as well as randomly rounding the result of a floating-point operation it can also randomly perturb its inputs and output.

### 8.4. Ordinary differential equations

The analysis of rounding errors in ODEs typically follows the classical convergence theory of time-stepping methods [29,83], in which the global error introduced by the ODE integration procedure is expressed in terms of the local errors introduced at each time step, and the global error is bounded in terms of the step size *h*, which we will assume is fixed. These local errors comprise both (local) truncation errors and (local) rounding errors. It is clear that unless the contribution to the global error from rounding errors decays to zero at the same rate as the contribution from truncation error, the overall convergence of the method can be impacted. Unfortunately, the analysis by Henrici [29,83] shows that the global error of an order-*p* ODE solver under RN is *O*(*uh*^−1^ + *h*^*p*^). The *O*(*uh*^−1^) term is often overlooked in the literature, and indeed in binary64 arithmetic the unit round-off *u* is usually small enough to make it negligible for the step sizes *h* of interest, but the *O*(*uh*^−1^) term cannot be neglected when computations are performed in reduced precision arithmetic.

While not explicitly mentioning SR, early work by Henrici in the 1960s [29,83] and by Arató in the 1980s [84] considers rounding errors arising in ODE solvers as independent (rather than mean-independent) random variables of zero mean. Henrici indicates that, whenever rounding errors have this random structure, the term *O*(*u*Δ*t*^−1^) can be replaced with a term characterized by a milder growth in Δ*t*. The analysis by Arató in [84] rewrites the problem of estimating the global rounding error as the solution of a stochastic differential equation. It is curious that stochastic differential equations have not yet appeared in the actual analysis of SR errors for ODEs and PDEs.

It has been shown experimentally that SR can alleviate the accumulation of rounding errors in ODE solvers. Hopkins *et al.* [39] and Mikaitis [40] use, on an ODE that models neurons in two configurations, four different solvers including RK2 Midpoint and RK3 Heun. They compare the results obtained in fixed-point arithmetic with those obtained using the same solvers run in binary32 and binary64 arithmetics. For the fixed-point solvers, they consider three rounding variants in the multiplication operation: bit truncation, RN and mode 1 SR. In the experiments, 32-bit fixed-point arithmetic with SR in multipliers shows accuracy similar to that of binary64 arithmetic in all cases, while fixed-point arithmetic with RN and bit truncation, as well as binary32 arithmetic, accumulate significant errors in the progression of the ODE system, ending up with a very different neuron behaviour.

**Figure 6.**
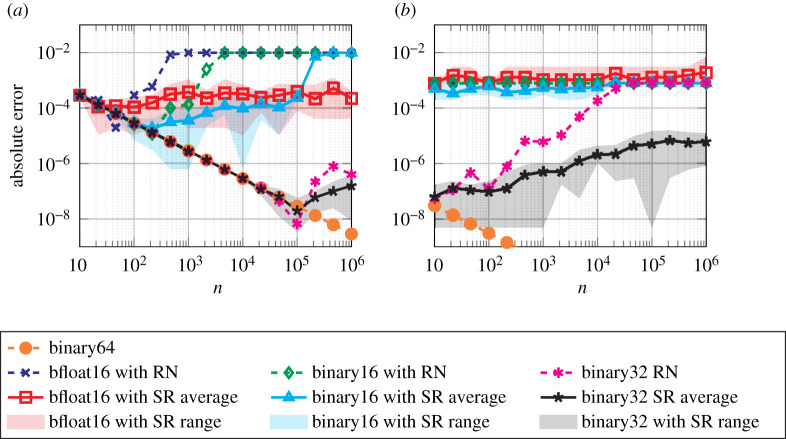
Absolute errors in the forward Euler method for an ODE with exponentially decaying solutions with different floating-point arithmetics and rounding modes. Stochastic rounding experiments are repeated 10 times; the solid line represents the average error, the edges of the shaded area the minimum and maximum error. The step size is the interval length divided by *n*. The experiment is adapted from [37]. (*a*) *y*′ = −*y*, *y*(0) = 2^−6^, over [0, 1], (*b*) $y^{\prime }=-y/20,y(0)=1\;\;\mathrm{over}\;[0,2^{-6}].$

Floating-point arithmetics (binary16, bfloat16, binary32) with SR in adds and multiplies have been considered by Fasi & Mikaitis [37]. ODEs exhibiting exponential decay were solved with Euler, midpoint and Heun solvers. For very small time steps, where rounding errors dominate the overall error of the solution, using SR produced a final solution error lower than that of RN. Figure 6 shows this for the solution errors with the forward Euler method solved in various arithmetics.

The ODE system

**Figure 7.**
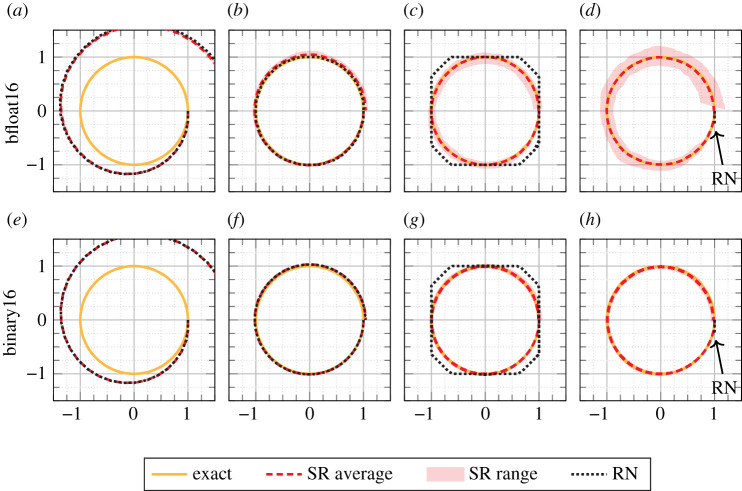
Computed solutions from the forward Euler method with SR and RN for the ODE system (8.4). The exact solution is the unit circle. The *x*- and *y*-axis represent *u* and *v*, respectively. Note that in (*d*) and (*h*) only a small part of the solution computed with round-to-nearest is visible (marked with an arrow) since the ODE solver failed because of stagnation. The experiment is adapted from [37]. Stochastic rounding experiments are repeated 10 times; the solid line represents the average trajectory, the edges of the shaded area the points that are farthest from the exact solution in the Euclidean distance. (*a*) *n* = 2^5^, (*b*) *n* = 2^9^, (*c*) *n* = 2^11^, (*d*) *n* = 2^13^, (*e*) *n* = 2^5^, (*f*) *n* = 2^9^, (*g*) *n* = 2^14^, (*h*) *n* = 2^16^.

8.4 $$\left\{ \begin{array}{cc} u^{\prime }(t)=v(t), & u(0)=1,\\ v^{\prime }(t)=-u(t), & v(0)=0, \end{array}\right.$$

whose solution is the unit circle in the (*u*, *v*) plane, was solved using binary16 and bfloat16 arithmetics for increasingly smaller integration steps [37, §8.3.2]. The results in figure 7 for the forward Euler method with *h* = 2*π*/*n* demonstrate that with RN the computed solutions are not meaningful for very small integration steps, while with SR the computed solution reproduces the unit circle quite well.

### 8.5. Partial differential equations

Little is known about the interplay between SR and the typical algorithmic components of PDE solvers, namely sparse iterative solvers, preconditioning, optimization and time-stepping methods.

Croci & Giles [43] analyse the effects of RN and SR in the solution of the heat equation with Runge–Kutta methods and finite differences, and explain how the numerical scheme should be implemented in order to reduce rounding errors. The analysis for RN yields the same *O*(*u*Δ*t*^−1^) rate in all dimensions as in Henrici’s work on ODEs [29,83], and in related work on the heat equation by Jézéquel [85]. On the other hand, using SR yields considerably smaller error bounds. In fact, Croci & Giles prove that the leading-order component of the rounding errors introduced by SR are zero-mean random variables that are independent in space and mean-independent in time. Thanks to this lack of correlation, much milder blow-up rates are obtained for the global rounding errors. These rates are essentially *O*(*u*Δ*t*^−1/4^) in one dimension (1D), *O*(*u*|log(Δ*t*)|^1/2^) in two dimensions (2D), and *O*(*u*) in three dimensions (3D). Interestingly, rounding errors become asymptotically smaller as the dimension increases: the larger the dimension, the more error cancellation occurs because of the spatial independence of the SR errors.

The lack of error correlation and the zero-mean property are not solely responsible for the success of SR for this problem. Croci & Giles also show that the RN solution is prone to stagnation, and in fact the phenomenon may occur from the very first step if Δ*t* is small enough to cause the RN solution to never move away from the initial condition. On the other hand, SR is resilient to stagnation, which did not affect the SR solution in numerical experiments.

In unpublished work, these results have been extended to linear parabolic PDEs and the finite-element method and, together with numerical experimentation in binary16 and bfloat16 precision, show that while RN can fail to compute meaningful solutions, SR computations always exhibit near-machine-precision accuracy for sufficiently small time steps and mesh sizes. We expect similar results as in the parabolic case to hold for hyperbolic PDEs, with the exception perhaps of the stagnation behaviour.

Here, we consider a diffusion equation with Dirichlet boundary conditions,

8.5 $$\left\{ \begin{array}{ccc} u_{t}(t,x)=\nabla \cdot (D(x)\nabla u(t,x))+f(x), & x\in D={\left(0,1\right)}^{d}, & t\in [0,1],\\ u(0,x)=u_{0}(x),\;u(t,s)=1,\; & s\in \partial D,\; & t\in [0,1], \end{array}\right.$$

where

$$D(x)=\left\{ \begin{array}{cc} \frac{1}{2}(\sin{\left(\pi x\right)}^{2}+1),\; & \text{in 1D},\\ \frac{1}{3}\left[\begin{array}{cc} \sin{\left(\pi x_{1}\right)}^{2}+1\; & \sin(\pi x_{1})\cos(\pi x_{2})\;\\ \sin(\pi x_{1})\cos(\pi x_{2})\; & \cos{\left(\pi x_{2}\right)}^{2}+1 \end{array}\right],\; & \text{in 2D},\; \end{array}\right.$$

and *f*(*x*) is chosen so that the exact solution to (8.5) at steady state is

$$u(\infty ,x)=\left\{ \begin{array}{cc} 16{\left(x(1-x)\right)}^{2}+1, & \text{in 1D},\\ {\left(16x_{1}x_{2}(1-x_{1})(1-x_{2})\right)}^{2}+1, & \text{in 2D}. \end{array}\right.$$

**Figure 8.**
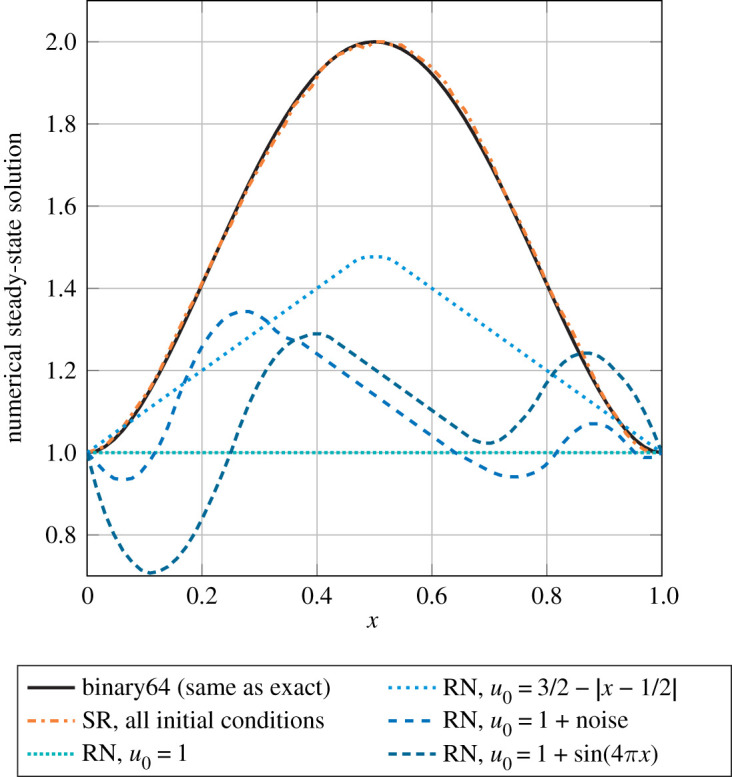
Comparison between the numerical steady-state solutions obtained with RN and SR with forward Euler and the bfloat16 format for different initial conditions. All SR solutions essentially converge to the same steady state. On the other hand, when RN is used different initial conditions lead to different steady-state solutions. The noise term in the initial condition has been obtained by sampling independent standard Gaussian random variables at each mesh node.

By using bfloat16 arithmetic with RN and SR, in figure 8 we show the effect of stagnation on the numerical steady-state solution of this problem in 1D as we vary the initial condition. We solve (8.5) using the finite-element method with piecewise linear basis functions and the forward and backward Euler schemes. We note that the RN solution always stagnates close to the initial condition, while SR successfully captures the correct steady-state solution.

**Figure 9.**
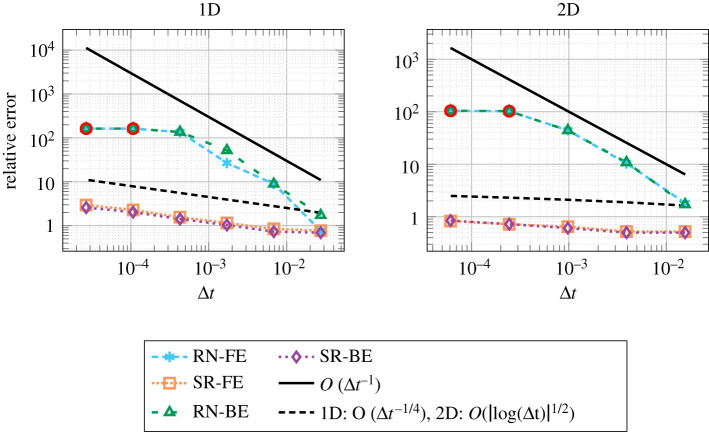
Plot of the relative global rounding error in the *L*^2^ norm for the solution of (8.5) in 1D (left) and 2D (right) with forward and backward Euler (FE and BE, respectively) and the bfloat16 format. We circled the RN data points for which the solution stagnates at the initial condition. The error behaviour matches the theoretical predictions from [43]. The SR errors are average errors computed within 2 digits of accuracy as in [43]. The worst-case SR errors were only a small constant factor larger than the average and are thus omitted.

In figure 9, we plot the relative (i.e. normalized by the unit round-off) global rounding errors for both RN and SR against the theoretical bounds of Croci & Giles [43]. While the RN error indeed grows linearly with Δ*t*^−1^ until stagnation, the SR error increases very mildly in 1D and almost unnoticeably in 2D. For a similar 3D problem (not shown), the errors are just bounded by a multiple of the unit round-off. It therefore seems that SR is able to control the growth of rounding errors without requiring more accurate summation techniques such as that in [86].

Paxton *et al.* [44] investigate experimentally the effects of RN and SR in chaotic ODE and PDE systems related to climate modelling: the Lorenz system, heat diffusion, a nonlinear shallow-water model for turbulent flow over a ridge, and a coarse resolution global atmospheric model with simplified parametrizations. They simulate these models in various precisions using from 62- to 10-bit floating-point formats and compare their results via the Wasserstein distance, a metric used to measure the discrepancy between probability distributions. They find that SR can effectively mitigate the growth of rounding errors in both simple heat diffusion and turbulent models. Furthermore, they report the occurrence of stagnation when RN is used to solve the heat equation, confirming the results in [43].

Overall, the findings by Paxton *et al.* show that reduced precision with SR is a valid alternative to standard binary64 precision computations. The authors also suggest that SR might become relevant in next-generation climate models.

### 8.6. Quantum mechanics

In quantum mechanics, an integer variant of SR has been used by several authors in order to estimate the dominant eigenvalues of Hamiltonian matrices using Monte Carlo versions of the power iteration. The goal is to compute the ground state eigenvector φ_0_ of a Hamiltonian matrix *H* as a linear combination of a set of *basis states* |0〉 , …, |*n*〉. The coefficients of the linear combination, or *basis-state amplitudes*, are the inner products *c*_*i*_ = 〈*i*|φ_0_〉. At each step of the power method, the coefficient *c*_*i*_ is approximated by an integer $n_{i}^{\left(k\right)}$, and for all *i* the approximations at step *k* + 1 are computed from those at step *k*. Once the iteration has converged numerically, the basis-state amplitude for the state |*i*〉 is estimated as the average value of $n_{i}^{\left(k\right)}$ over *k*.

Nightingale & Blöte [87] are the first to suggest the use of SR for the solution of this problem. They use a random-walk model, and in their work the integers $n_{i}^{\left(k\right)}$ count the number of random walkers that are in state |*i*〉 at iteration *k*. The integer SR function used in this work is

8.6 $$\mathrm{fl}(x)=\left\{ \begin{array}{cc} {}[x]+1, & \text{with probability }x-[x],\\ {}[x], & \text{with probability }1-(x-[x]), \end{array}\right.$$

where [*x*] denotes the integer part of x∈R, defined by

$$[x]=\left\{ \begin{array}{cc} \lfloor x\rfloor , & x\geq 0,\\ \lceil x\rceil , & x<0. \end{array}\right.$$

Allton *et al*. [88] improve on this idea by suggesting a new scheme, called *stochastic truncation*, further developed by Hamer *et al.* [89] and Hamer & Court [90]. All these variants of the stochastic truncation method use essentially the same rounding function (8.6).

Price *et al*. [91] propose a variation of (8.6) which essentially applies integer SR only to a specific interval

$$\mathrm{fl}(x)=\left\{ \begin{array}{cc} x, & x\geq 1,\;\;\\ 1, & 1>x>P,\\ 0, & P\geq x>0, \end{array}\right.$$

where *P* is a random number from the interval [0, 1]. This rounding operator keeps the ‘exact’ value of *x* for large *x* but allows some of the values below 1 to be stochastically replaced by 0.

### 8.7. Quantum computing

Krishnakumar & Zeng [46] show how to implement mode 1 and 2 SR for quantum computing applications and demonstrate that mode 1 provides accuracy or circuit complexity improvements. Mode 1 SR in this work is called *quantum rounding*. It is shown that quantum rounding can be implemented by using the fact that quantum computing has a probabilistic component—measuring a state of a quantum register can return different results with certain probabilities. The authors show that a quantum rounding circuit can be made to round with proportional probabilities according to the mode 1 stochastic rounding in (2.1). Once such a circuit is used multiple times to measure the value of a quantum register (as is commonly done in quantum computing in order to improve confidence in the results), the average value will be more accurate because of the properties of mode 1 SR. The authors show that implementing fixed-point multiplication in a fault-tolerant quantum setting requires two to three times less resources for the same accuracy targets, compared with when RN is used.

### 8.8. Other applications

Various other applications use SR in one way or another. We give overviews of a few such applications.

In digital signal processing, SR goes under the name *random rounding* and has been considered for fixed-point arithmetic. Callahan [92] demonstrates a simple 16-bit filter that is more accurate with SR than with the standard rounding. Two hardware implementations of SR are also demonstrated, and one of them interestingly does not require random number generation but uses a value that is perturbed on each rounding operation.

Bargh *et al.* [93] and Tran *et al.* [94] address the problem of preserving privacy when publicly releasing datasets. Their goal is to find the best ways to minimize the disclosure of personal information and share only data that does not infringe peoples’ privacy. One of the aspects considered is how to transform sensitive information in specific cells of tabulated data. In [93, §4.2.2], rounding is discussed as an alternative to suppression, which is the simple removal of values that are at risk of disclosing private information, a process which may potentially delete useful data. In this research SR, under the name of *random rounding* [95, §5.4.3], is used to round numerical data to one of the two nearest integer multiples of a given base. In base 10, for example, the number 26 would be rounded to 20 or to 30 with probabilities 40% and 60%, respectively. SR is useful here as it does not always increase large values and decrease small ones as RN would [93]. Being unbiased, moreover, SR can hide the information about the original data [95], and may even provide protection against *differencing*, where sensitive information can be extracted from the differences in multiple tables [95].

Rounding to integer in a stochastic fashion is also considered by Gösgens *et al.* [96] in the study of models for the spread of infections, by Matter & Potgieter [97], to solve a problem of resource allocation, and by Hörl & Balac [98], for exploring travel demand in cities using transport simulations.

Wu [99] explores SR and a modification of it called *dither rounding* in the context of stochastic computing. Dither rounding is more complex than SR, as it requires keeping track of the number of rounding operations performed. However, Wu shows that dither rounding can achieve similar accuracy, but with lower variance, in matrix multiplication and in machine learning algorithms for digit classification.

There is some connection between SR and the technique of *dither* that is a common component in audio analogue-to-digital and digital-to-analogue conversion [100–102]. In analogue-to-digital conversion, dither relates to the randomization the analogue signal undergoes before being converted to a low-precision quantized digital representation [101]. The same term is used by the authors to refer to the randomization at the other end, when converting a digital signal back to analogue. For example, the following excerpt from [101] discusses a method of dither of the digital signal that is similar to an implementation of SR mode 1 where a set of random bits are added to the part of fraction to be truncated.

*If a digital manipulation (such as a gain reduction) is performed, there may be a tendency to take the intermediate higher precision numbers generated by the multiplication and simply truncate or round them to the bit width of the system. This will in many cases leave the signal improperly dithered. [···] The fractional truncated bits have some influence on the dither, in keeping with their relative position. If cost or processing time were no object, then any digital manipulation should be carried out with full accuracy, and the dither carry bit (0 or 1) can be determined by an appropriate digital random number added to the bits to be truncated. In practice such schemes would probably work well by considering only the first 3 or 4 bits to be truncated.*

See also [101, fig. 9] for a diagram that sketches an implementation of SR mode 1 in an integer multiplier.

## 9. Conclusion

Hardware units supporting SR are not yet widely available but have started to appear: as we discussed in §7.5, Graphcore and Intel are producing processors with SR built in. Patents from AMD, NVIDIA, IBM and other computing companies describing implementations of SR in fixed- or floating-point arithmetic units show that this rounding mode could become more widely available in the future.

When hardware is not available, simulation in software of arithmetics with SR can be used to explore its behaviour. Multiple simulators have been developed, as discussed in §7.4. These are available in various forms for Matlab, C, Julia and Python.

Rounding error analysis with SR, discussed in §6, shows that compared with the standard rounding modes, SR guarantees probabilistic error bounds significantly smaller than the worst-case bounds and it also avoids the problem of stagnation (§6.1), where small values are lost to rounding when they are added to an increasingly large accumulator. This explains the success of SR in the applications described in §8.

We covered work using SR in various forms, in numerical linear algebra, machine learning, ODE and PDE solvers, quantum computing and other areas. The wide array of applications in which SR has been tried and led to improved accuracy demonstrates that it is a useful technique to consider when arithmetics with standard rounding modes are insufficiently accurate. SR provides a useful alternative to extended-precision registers, arbitrary-precision libraries, multi-word representations and arithmetics, compensated algorithms and other means for improving accuracy.

## Data Availability

Data and relevant code for this research work are stored in GitHub: https://github.com/mmikaitis/stochastic-rounding-survey-experiments and have been archived within the Zenodo repository: https://doi.org/10.5281/zenodo.5983596.

## Footnotes

1 Electronic numerical integrator and computer—the first programmable, general-purpose digital computer, made in 1945.

2 We remark that floating-point numbers are not uniformly distributed in [0, 1], but here we need the precision-*k* random floating-point number to be sampled from that interval uniformly in the sense of real numbers. This is not a consideration required in the hardware algorithms in §7.3 since there SR is performed at the bit level, using uniformly distributed integers. Usage of other random number distributions in SR has been explored by Xia *et al.* [36].

3 See https://github.com/higham/chop.

4 See https://gerard-meurant.pagesperso-orange.fr/floatp.zip.

5 See https://github.com/Tiiiger/QPyTorch.

6 See https://github.com/mfasi/cpfloat.

7 See https://github.com/verificarlo/verificarlo.

8 See https://github.com/edf-hpc/verrou.

9 See https://github.com/milankl/StochasticRounding.jl.

10 See https://juliarandom.github.io/RandomNumbers.jl/stable/man/xorshifts/.

11 See https://github.com/SpiNNakerManchester/spinn_common/blob/master/include/round.h.

12 See https://github.com/mmikaitis/stochastic-rounding-survey-experiments.
